# *Mycobacterium avium* subsp. *hominissuis* Infection in a Pet Parrot

**DOI:** 10.3201/eid1504.081003

**Published:** 2009-04

**Authors:** Edmealem Jembere Shitaye, Veronika Grymova, Martin Grym, Roman Halouzka, Alica Horvathova, Monika Moravkova, Vladimir Beran, Jana Svobodova, Lenka Dvorska-Bartosova, Ivo Pavlik

**Affiliations:** Veterinary Research Institute, Brno, Czech Republic (E.J. Shitaye, A. Horvathova, M. Moravkova, V. Beran, L. Dvorska-Bartosova, I. Pavlik); University of Veterinary and Pharmaceutical Sciences, Brno (E.J. Shitaye, R. Halouzka); Veterinary Clinic AvetuM, Brno (V. Grymova, M. Grym); Regional Institute of Public Health, Brno (J. Svobodova)

**Keywords:** Mycobacteria, tuberculosis, Mycobacterium avium, zoonoses, birds, parrot, pets, letter

**To the Editor:** Tuberculosis is a chronic wasting disease in domestic birds (especially hens) and free-ranging birds worldwide (*1*). Most mycobacterial infections in birds are caused by *Mycobacterium avium* subsp. *avium* (mainly domestic birds) or by *M. genavense* (especially pet birds). Nontuberculous (potentially pathogenic) mycobacteria (i.e., *M. fortuitum*, *M. gordonae*, and *M. nonchromogenicum*) occasionally have been isolated from necropsied pet birds (*2*). Because potentially pathogenic mycobacteria also are increasingly problematic in immunocompromised human patients, they merit special attention. *M. avium* subsp. *hominissuis* can infect humans, especially immunocompromised persons. *M. avium* subsp. *hominissuis* infections have been documented in pigs and cattle (*3*) and rarely in dogs (*4*), birds (*5*), and other animals.

We report a case of *Mycobacterium* infection in a female blue-fronted Amazon parrot (*Amazona aestiva*; pet bird) ≈6 months of age that was brought to a clinic because of inappetence over a 3-day period and polydipsia and yellow coloration of urine. Clinical examination showed slight emaciation, heavy biliverdinuria, ascites, and melena. By coprologic examination, 3 eggs of *Ascaridia* sp. worms were found in 1 field of view using 40× magnification. A Gram stain of fecal material showed sporadic gram-positive rods. On the basis of these signs, chlamydiosis was suspected. Differential diagnosis suggested liver cirrhosis, neoplasia, and Pacheco disease. Enrofloxacin (Baytril 2.5% injectable; Bayer AG, Frankfurt am Main, Germany) was administered subcutaneously (0.15 mL injected subcutaneously) and albendazole (Aldifal 2.5% suspension; Mevak a.s., Nitra, Slovakia) were administered orally (0.2 mL injected subcutaneously). The bird died 1 day later.

Necropsy showed ascites (clear yellowish fluid), hepatomegaly (stiff liver consistency, yellow-pink), mild splenomegaly, and hemorrhagic enteritis with thickening of the intestinal wall; the finding of hemorrhagic enteritis was unclear because the intestinal mucosa was hyperemic and covered with a thick layer of viscous mucus that contained blood. Twenty worms (*Ascaridia* sp.) were observed in the intestinal lumen.

Histopathologic examination showed diffused liver fibrosis with cystic dilatation of the bile ducts and focal extramedullary hematopoiesis. The hepatic parenchyma was nearly completely atrophic. Only some clusters of atrophic hepatocytes were observed. The other organs (kidneys, spleen, lungs, brain, and intestines) were free of histopathologic lesions. Hypertrophic cirrhosis (chronic active hepatitis) was diagnosed. Neither granulomatous nor other lesions were observed.

After Ziehl-Neelsen stain of tissue impressions, acid-fast rods (AFRs) were microscopically detected in the liver and intestine. Cultivation according to Matlova et al. (*6*) grew 6 acid-fast rod–positive isolates from 9 examined tissue specimens. A PCR assay confirmed *M. avium* spp., and a subsequent PCR assay for *M. avium* differentiation indicated *M. avium* subsp. *hominissuis* (IS*1245*+ and IS*901–*); both PCR assays were performed as described (*7*). The *M. avium* subsp. *hominissuis* isolate was classified as serotype 9. Typing of all isolates by IS*1245* restriction fragment length polymorphism (RFLP) analysis according to Van Soolingen et al. (*8*) showed 2 different multibanded IS*1245* RFLP types, which varied in only 1 band position (Table).

**Table:** Detection of *Mycobacterium avium* subsp. *hominissuis* in a female blue-fronted Amazon parrot (*Amazona aestiva*)*

Origin of examined tissue samples	Mycobacteria detection			PCR†		IS*1245* RFLP§
	ZN	Culture‡		IS*1245*	IS*901*	
Lung	–	+ (3)		+	–	a
Kidney	–	–				
Heart	–	–				
Liver	+	+ (15)		+	–	a
Intestine	+	+ (2)		+	–	b
Stomach	–	+ (8)		+	–	a
Bone marrow	–	+ (1)		+	–	a
Brain	–	–				
*Musculus pectoralis*	–	+ (34)		+	–	a

*ZN, Ziehl-Neelsen microscopy of homogenate for acid-fast rods; RFLP, restriction fragment length polymorphism; –, not detected/negative; +, detected/positive. †PCR for IS*1245* and IS*901* was carried out according to the method described in (*7*). ‡Culture examination performed as described by Matlova et al. (*6*); colony-forming units per isolation are shown in parentheses. §Standardized IS*1245* RFLP method was performed according to Van Soolingen et al. (*8*).

*M. avium* subsp. *hominissuis* is not considered an avian pathogen and rarely has been isolated from tuberculous lesions (*5*). However, our case study reports the isolation of *M. avium* subsp. *hominissuis* from multiple organs of 1 exotic bird that had developmental anomaly and liver fibrosis (Table). In addition to a few nonspecific gross lesions, nontuberculous lesions were observed in the liver, spleen, and intestinal organs.

The etiology of mycobacteriosis, especially in pet birds, is rarely identified. This may be because intravitam and postmortem findings are nonspecific. Infection with *M. avium* subsp. *hominissuis* may not lead to tuberculous lesions in birds, particularly when the infection occurs without complications. Susceptibility to mycobacterial infection, including *M. avium* subsp. *hominissuis* , depends on the host’s immune and nutritional status, environmental conditions unfavorable for the host, and genetic factors (*1*,*9*). Consistent with these reports, in this case, the histologic findings such as fibrosis of the liver associated with cystic dilatation and intestinal ascaris infestation may have aggravated the intensity of the mycobacterial infection.

IS*1245* RFLP analysis showed isolates with 2 profiles that differ in the presence of only 1 band. The additional band in the rest of the isolates probably represents the transpositional event. The variability in 1 or 2 bands of 1 strain was also observed previously (*10*); therefore, we presume the bird was infected by only 1 strain of *M. avium* subsp. *hominissuis* . Unfortunately, the source of infection for this bird was not identified.

A multibanded IS*1245* RFLP profile was described in a *M. avium* isolate from a parrot (*4*), but no details about this case were given. Our findings suggest that owners of pet birds and their family members may be at risk from this pathogenic causal agent. Hence, immunocompromised persons, children, and others involved in the breeding of exotic birds should avoid contact with birds with clinically suspected *M. avium* subsp. *hominissuis* .
